# Association of Anthropometric Obesity Measures With Semen Parameters in Male Partners of Infertile Couples Without Identifiable Clinical Risk Factors

**DOI:** 10.7759/cureus.104455

**Published:** 2026-02-28

**Authors:** Onkar B Waghmare, Nadia Sandhu, Sajidali S Saiyad, Jaskirat K Hundal, Roma Umakant Dubey, Padmavathi Parthasarathy, Gnanadesigan Ekambaram, Shivani U Parikh, Sudhir Prabhakar Choudhari

**Affiliations:** 1 Department of Physiology, Pacific Medical College and Hospital, Pacific Medical University, Udaipur, IND; 2 Department of Clinical Sciences, Washington University of Health and Science, San Pedro, BLZ; 3 Department of Surgery, American University of Antigua, Chicago, USA; 4 Department of Obstetrics and Gynaecology, Aryavart Hospital, Meerut, IND; 5 Department of Biochemistry, Rajalakshmi Medical College Hospital and Research Institute, Pennalur, IND; 6 Department of Physiology, Nootan Medical College and Research Centre, Sankalchand Patel University, Visnagar, IND; 7 Department of Obstetrics and Gynaecology, Parul Institute of Homeopathy and Research, Parul University, Waghodiya, IND; 8 Department of Physiology, Goverment Medical College Chhatrapati Sambhaji Nagar, Chhatrapati Sambhaji Nagar, IND

**Keywords:** anthropometric indices, body fat distribution, central obesity, semen quality, s : male infertility, sperm motility, waist-to-height ratio

## Abstract

Background: Male infertility is an increasingly recognized contributor to the global infertility burden, occurring alongside reported declines in semen quality and a rising prevalence of obesity, particularly central obesity, in many populations. Although adiposity and body fat distribution have been implicated in male reproductive health, existing evidence remains inconsistent and often relies solely on body mass index.

Objective: To evaluate the association between general and central anthropometric measures of obesity and semen parameters in male partners of infertile couples without identifiable clinical risk factors for infertility other than adiposity.

Methods: This hospital-based cross-sectional analytical study included 100 male partners of infertile couples aged 21-50 years. Anthropometric assessment comprised body mass index, waist circumference, waist-hip ratio, and waist-height ratio using population-appropriate cut-offs. Semen analysis was performed in accordance with World Health Organization 2010 guidelines. Group comparisons were conducted using non-parametric methods, and associations were assessed using Spearman's rank correlation, with adjustment for multiple comparisons.

Results: Higher levels of general and central adiposity were associated with poorer semen quality parameters. Men with elevated body mass index demonstrated lower sperm concentration, total sperm count, total motility, and progressive motility compared with those with normal body mass index, while semen volume showed minimal variation. Measures of central obesity exhibited more moderate inverse associations with semen parameters than body mass index alone. Across all anthropometric indices, progressive sperm motility showed the most pronounced inverse association, whereas semen volume remained largely unaffected.

Conclusions: Both general and, more prominently, central adiposity are associated with adverse semen quality profiles, particularly with respect to sperm motility. Incorporation of waist-based anthropometric indices alongside body mass index may enhance risk characterization among male partners of infertile couples. Prospective and interventional studies are required to clarify causality and to determine whether modification of central adiposity is associated with improvements in semen quality and reproductive outcomes.

## Introduction

Infertility is defined as the failure to achieve a clinical pregnancy after 12 months or more of regular, unprotected sexual intercourse and is classified as a disease of the reproductive system in the International Classification of Diseases, 11th Revision (ICD-11) [1]. Although not life-threatening, infertility imposes substantial psychological, social, and economic burdens on affected individuals and couples. Male factors are increasingly recognized as major contributors to infertility, accounting for a significant proportion of cases either independently or in combination with female factors [2]. This shift in perspective has highlighted the need to identify modifiable determinants of male reproductive dysfunction.

Over recent decades, global and regional evidence has demonstrated a concerning decline in semen quality. Reductions in key semen parameters, including sperm concentration and morphology, have been widely reported, suggesting a deterioration in male reproductive potential [2,3]. In India, longitudinal analyses indicate a significant decline in seminal quality over the past 37 years, raising concerns regarding male reproductive health in populations undergoing rapid lifestyle and nutritional transitions [3]. These trends point toward an increasing influence of environmental and metabolic factors on male fertility. 

Concurrently, obesity has emerged as a major global health challenge. The World Health Organization (WHO) reports a dramatic rise in the prevalence of overweight and obesity across all age groups and regions [4]. Obesity is characterized by excessive fat accumulation associated with adverse metabolic and endocrine consequences [5]. Importantly, WHO expert consultations have emphasized that obesity-related health risks are determined not only by total body fat but also by fat distribution, particularly central or abdominal adiposity [5,6]. 

Accumulating evidence implicates obesity as a potentially modifiable risk factor for male infertility. Excess adiposity has been associated with hormonal dysregulation, altered testosterone-to-estradiol ratios, impaired spermatogenesis, and compromised semen quality [7,8]. Consequently, numerous studies have explored the relationship between obesity and semen parameters, most commonly using body mass index (BMI) as a surrogate measure of adiposity.

Several investigations have demonstrated inverse associations between BMI and semen quality, reporting reductions in sperm concentration, total sperm count, and motility with increasing BMI [9-11]. Systematic reviews and meta-analyses further support these findings, indicating a higher prevalence of oligozoospermia and azoospermia among overweight and obese men [7]. However, other studies have failed to demonstrate consistent relationships between BMI and semen parameters or reproductive hormone levels, highlighting substantial heterogeneity in the existing literature [7,8].

A key limitation of BMI is its inability to distinguish between lean and fat mass or to reflect regional fat distribution. Recognizing this limitation, WHO expert groups have recommended the inclusion of additional anthropometric measures, such as waist circumference (WC) and waist-to-hip ratio (WHR), to better assess obesity-related health risks [5,6]. These measures more accurately capture central adiposity, which is metabolically active and closely linked to endocrine and inflammatory pathways relevant to reproductive function.

Recent studies suggest that indices of central obesity may be more strongly associated with semen quality than BMI alone. Eisenberg et al. reported significant inverse associations between waist circumference and semen volume, sperm concentration, and total sperm count [9]. Fejes et al. demonstrated negative correlations between body fat distribution indices and sperm count, motility, and semen volume [8], while Keszthelyi et al. showed that the waist-to-hip ratio may better predict impaired semen parameters than BMI [9]. Nevertheless, inconsistencies persist, with some analyses reporting weak or non-significant associations, likely reflecting differences in study populations, methodologies, and confounding factors [7,8].

Ethnic and population-specific considerations further complicate interpretation. WHO expert consultations have emphasized that Asian populations experience higher metabolic risk at lower BMI thresholds compared with Western populations [12], and lower waist circumference cut-offs have been recommended for Asian Indians to identify abdominal obesity more accurately [13]. The waist-to-height ratio (WHtR) has also emerged as a simple and effective screening tool for obesity-related risk [14], yet its relationship with semen parameters remains inadequately explored.

Importantly, obesity-related impairments in semen quality may be reversible. Improvements in semen parameters and reproductive hormone profiles following weight loss have been reported, supporting a potential causal relationship between adiposity and male reproductive dysfunction [15]. However, data from Indian and Asian populations remain limited, particularly studies that simultaneously evaluate general and central obesity using multiple anthropometric indices in male partners of infertile couples without identifiable clinical or surgical risk factors for infertility, assessed using standardized semen analysis protocols [16].

This study systematically evaluates the relationship between multiple anthropometric measures of obesity, including BMI, waist circumference, waist-to-hip ratio, and waist-to-height ratio, and semen parameters in men with no known risk factors for infertility. By integrating measures of both general and central obesity and applying population-appropriate cut-offs, this work moves beyond BMI-centric assessments to provide a more refined understanding of the association between adiposity and male reproductive health. The findings are expected to clarify inconsistent evidence, enhance early risk stratification, and inform preventive and lifestyle-based strategies aimed at preserving male fertility.

## Materials and methods

Study design and setting

This was a hospital-based cross-sectional analytical study conducted at the Reproductive Biology Unit of the Department of Physiology in a government tertiary care hospital in Aurangabad (Chhatrapati Sambhaji Nagar), Maharashtra, India. Participant recruitment and data collection were carried out from December 2020 to June 2023. Data were analyzed subsequently, and the manuscript was prepared after completion of data collection. A cross-sectional design was selected, as it is appropriate for evaluating associations between anthropometric indices of obesity and semen parameters at a single time point in a defined population.

Ethical considerations

The study protocol was reviewed and approved by the Institutional Ethics Committee (IEC/IRB) - Govt. Medical College, Aurangabad, with proposal number Pharma/IEC-GMCA/3/2020 prior to initiation (approval date: 12 November 2020). The study was conducted in accordance with the Declaration of Helsinki. Written, valid, and informed consent was obtained from all participants in a language they could understand before enrollment.

Study population and recruitment

The study population consisted of male partners of infertile couples attending the reproductive biology unit for routine semen analysis. Participants were recruited consecutively and included only if they had no identifiable clinical, surgical, endocrine, or lifestyle-related risk factors for infertility other than increased adiposity, based on detailed history and physical examination.

Participants were eligible for inclusion if they were male partners of infertile couples aged between 21 and 50 years who presented to the reproductive biology unit for semen analysis and were willing to participate voluntarily after providing written informed consent. Participants were included only if no identifiable non-obesity-related risk factors for male infertility were present.

To minimize confounding and isolate the effect of obesity on semen parameters, individuals with known risk factors other than obesity that could influence sperm quality were excluded from the study. These exclusion criteria included the presence of varicocele or a history of varicocelectomy within the preceding six months, previous scrotal surgery such as orchiopexy for undescended testis, testicular tumors, or a solitary testis. Participants with known endocrine disorders, including diabetes mellitus, hypothyroidism, hyperthyroidism, hypogonadism, or hyperprolactinemia, were also excluded. Additional exclusions comprised genital tract infections such as orchitis, epididymitis, or urethritis; neurological or psychological disorders; current use of medications or chemotherapy; alcohol or tobacco addiction; and semen diagnoses of azoospermia.

Sample size calculation

The sample size was determined based on the primary objective of detecting associations between anthropometric indices and semen parameters. For a correlational study design, a minimum sample size of 85 participants provides 80% power to detect a moderate correlation coefficient (ρ = 0.30) using a two-tailed significance level of α = 0.05. To allow for subgroup analyses, enhance statistical robustness in the presence of non-normal data distributions, and reduce the impact of potential data attrition, a target sample size of 100 participants was adopted.

Following data collection, the observed correlation between body mass index and progressive sperm motility (r = −0.43) was consistent with the effect size assumptions used for planning, indicating that the achieved sample size was sufficient to detect clinically meaningful associations. All inferential analyses incorporated appropriate non-parametric methods and adjustments for multiple comparisons.

Data collection and baseline assessment

Eligible participants were assigned a unique study identification number, and a structured case record form (CRF) was used to collect baseline data. A detailed history was obtained, including demographic characteristics, medical and surgical history, lifestyle factors, dietary habits, and treatment history. A general and systemic physical examination was performed to reconfirm eligibility.

Anthropometric measurements

All anthropometric measurements were performed by trained personnel using standardized procedures.

Body weight and height

Body weight was measured in kilograms using a calibrated electronic weighing scale with participants wearing minimal clothing. Height was measured in meters using a wall-mounted stadiometer with participants barefoot, standing erect with heels against the wall.

Body mass index

BMI was calculated as: body mass index (BMI) = weight (kg) / [height (m)]²

BMI classification followed Asian Indian cut-offs: normal (18.0-22.9 kg/m²), overweight (23.0-24.9 kg/m²), and obesity (≥25 kg/m²) [14].

Waist and hip circumference

Waist circumference was measured at the midpoint between the lower margin of the last rib and the iliac crest at the end of normal expiration using a constant-tension tape. Hip circumference was measured at the widest point over the buttocks.

Indian cut-off values for waist circumference were applied to define central obesity [15].

Waist-to-hip ratio and waist-to-height ratio

WHR was calculated as waist circumference divided by hip circumference, with a cut-off value of ≥0.9 indicating central obesity [16]. WHtR was calculated as waist circumference divided by height, with a cut-off of 0.5, as recommended for universal risk stratification [17].

Semen collection and analysis

Semen analysis was performed in accordance with the WHO Laboratory Manual for the Examination and Processing of Human Semen (5th edition) [13].

Sample collection

Participants were instructed to maintain sexual abstinence for 2-7 days prior to sample collection. Semen samples were collected by masturbation into sterile wide-mouthed containers in a private room near the laboratory during morning hours. Samples were allowed to liquefy for 30 minutes and protected from temperature extremes.

Macroscopic examination

Macroscopic parameters assessed included liquefaction time, viscosity, appearance, and semen volume. Semen volume was measured directly using graduated containers (accuracy 0.1 mL).

Microscopic examination

Semen analysis was performed using a phase-contrast microscope and a 10-µm depth counting chamber. Samples were mixed gently before analysis. Parameters assessed included sperm concentration, total sperm count, motility (progressive, non-progressive, and immotile), aggregation, agglutination, and presence of round cells.

Sperm motility was assessed within one hour of ejaculation. A minimum of 200 spermatozoa across multiple fields was evaluated per sample to minimize sampling error. Reference limits followed WHO recommendations.

Statistical analysis

Data were entered into a spreadsheet and analyzed using IBM Corp. Released 2019. IBM SPSS Statistics for Windows, Version 25. Armonk, NY: IBM Corp. Continuous variables were expressed as mean ± standard deviation or median (interquartile range) based on distribution. Data distribution for continuous variables was assessed visually and analytically and demonstrated non-normality, as evidenced by wide ranges and high variability across several semen parameters. Accordingly, non-parametric statistical methods were applied.

Given the multiple comparisons performed across anthropometric indices and semen parameters, adjustment for multiple testing was applied to control the risk of type I error. Statistical significance was interpreted using the Holm-Bonferroni method, which provides a balance between type I error control and statistical power for correlated outcomes.

Comparisons between two independent groups (normal vs. increased anthropometric categories) were performed using the Mann-Whitney U test, while comparisons across more than two body mass index categories were conducted using the Kruskal-Wallis test.

To explore potential confounding, exploratory multivariable analyses were conducted, adjusting for age and abstinence duration, which are known to influence semen parameters. Given the modest sample size, these analyses were considered supportive and hypothesis-generating rather than confirmatory.

Associations between anthropometric indices and semen parameters were assessed using Spearman’s rank correlation coefficient. Continuous variables were summarized as median (interquartile range), and a two-tailed p-value < 0.05 was considered statistically significant.

For categorical analyses, “normal” and “increased” anthropometric categories were defined according to Asian Indian cut-offs (BMI ≥23 kg/m²; WC ≥90 cm; WHR ≥0.90; WHtR ≥0.50). “Abnormal” semen parameters were defined according to WHO 2010 lower reference limits (semen volume <1.5 mL, sperm concentration <15 million/mL, total sperm count <39 million, total motility <40%, progressive motility <32%).

## Results

All reported p-values reflect adjustment for multiple comparisons unless otherwise specified.

Baseline anthropometric and semen characteristics

A total of 100 participants were included in the analysis. Baseline anthropometric measurements and semen characteristics are presented in Table 1. Participants showed considerable variability across body composition indices and semen parameters, enabling robust comparative and correlational analyses. Mean BMI indicated an overall tendency toward overweight, while semen parameters demonstrated a wide physiological range.

**Table 1:** Distribution of participants according to anthropometric and semen parameter categories (n = 100) BMI: Body mass index; WHR: Waist–hip ratio; WHtR: Waist–height ratio. Reference ranges are provided where applicable. † Anthropometric cut-offs based on Asian Indian population criteria. ‡ Reference ranges for semen parameters based on the WHO laboratory manual for the examination and processing of human semen, 6th edition [13].

Variable	Reference Range(Where applicable)*	Mean ± SD	Median (Range)
Anthropometric measures			
Age (years)	21–50	30.02 ± 5.32	29 (21–49)
Weight (kg)	—	66.42 ± 13.51	64.0 (45.0–106.0)
Height (cm)	—	166.39 ± 5.85	167.0 (145.0–180.0)
Body mass index (kg/m²)	18.5–22.9†	23.93 ± 4.65	23.0 (17.14–37.55)
Waist circumference (cm)	<90†	86.87 ± 12.33	86.5 (65.0–125.0)
Hip circumference (cm)	—	90.48 ± 9.32	90.0 (70.0–120.0)
Waist–hip ratio	<0.90†	0.95 ± 0.07	0.97 (0.79–1.14)
Waist–height ratio	<0.50	0.51 ± 0.07	0.53 (0.36–0.71)
Semen parameters			
Semen volume (mL)	≥1.5‡	3.10 ± 1.52	3.0 (0.5–8.0)
Sperm concentration (million/mL)	≥15‡	63.08 ± 53.54	50.0 (1.0–200.0)
Total sperm count (million/ejaculate)	≥39‡	204.80 ± 200.08	145.0 (1.0–750.0)
Total motility (%)	≥40‡	45.45 ± 31.19	60.0 (0–90)
Progressive motility (%)	≥32‡	31.04 ± 26.03	30.0 (0–86)

Distribution of anthropometric and semen parameter categories

The distribution of participants according to anthropometric and semen quality classifications is summarized in Table 2. While approximately half of the participants were categorized as overweight or obese based on BMI, a greater proportion exhibited central obesity when assessed using the waist-hip ratio and the waist-height ratio. Among semen parameters, progressive motility abnormalities were the most prevalent, whereas semen volume remained normal in most participants.

**Table 2:** Distribution of participants according to anthropometric and semen parameter categories (n = 100) Legend: Data presented as number (percentage). BMI: Body mass index; WC: Waist circumference; WHR: Waist–hip ratio; WHtR: Waist–height ratio. Semen parameter reference values are based on World Health Organization criteria. No inferential statistical comparisons were performed for categorical distributions.

Variable	Normal n (%)	Abnormal n (%)	Total
Anthropometric categories			
Body mass index	50 (50%)	50 (50%)	100
Waist circumference	55 (55%)	45 (45%)	100
Waist–hip ratio	22 (22%)	78 (78%)	100
Waist–height ratio	39 (39%)	61 (61%)	100
Semen parameters			
Semen volume	87 (87%)	13 (13%)	100
Sperm concentration	71 (71%)	29 (29%)	100
Total sperm count	72 (72%)	28 (28%)	100
Total motility ≥ 40%	60 (60%)	40 (40%)	100
Progressive motility ≥ 32%	49 (49%)	51 (51%)	100

Comparison of semen parameters across BMI and waist circumference categories

Comparisons of semen parameters across body mass index and waist circumference categories are presented in Tables 3, 4. Given the non-normal distribution of semen parameters, results are expressed as median (interquartile range), and group comparisons were performed using non-parametric methods.

**Table 3:** Comparison of semen parameters across body mass index using non-parametric analysis Values are expressed as median (interquartile range). Group comparisons were performed using the Mann–Whitney U test. The U value represents the test statistic based on ranked observations from the two independent groups; smaller U values indicate greater separation between group distributions. p-values were adjusted for multiple comparisons using the Holm–Bonferroni method. Statistical significance was defined as an adjusted p-value < 0.05. BMI: body mass index.

Parameter	Median (IQR)- Body mass index <23 kg/m²	Median (IQR)- Body mass index ≥23 kg/m²	U value	Adjusted p-value†
Semen volume (mL)	3.0 (2.0–4.0)	2.5 (1.5–4.0)	1150.5	0.331
Sperm concentration (million/mL)	80 (40–120)	25 (10–80)	850	0.001
Total sperm count (million/ejaculate)	300 (150–400)	60 (10–200)	812.5	0.001
Total motility (%)	65 (45–80)	25 (5–60)	755	0.001
Progressive motility (%)	40 (20–60)	10 (0–40)	720.5	0.001

**Table 4:** Comparison of semen parameters across waist circumference categories using non-parametric analysis Values are expressed as median (interquartile range). Group comparisons were performed using the Mann–Whitney U test. †P values were adjusted for multiple comparisons using the Holm–Bonferroni method. Statistical significance was defined as adjusted p < 0.05. The U value represents the test statistic based on ranked observations from the two independent groups; smaller U values indicate greater separation between group distributions.

Parameter	Median (IQR) - Waist circumference <90 cm	Median (IQR) - Waist circumference ≥90 cm	U value	Adjusted p-value†
Semen volume (mL)	3.0 (2.0–4.0)	2.5 (1.5–4.0)	1205	0.165
Sperm concentration (million/mL)	70 (30–120)	40 (10–80)	1050	0.007
Total sperm count (million/ejaculate)	250 (120–400)	140 (20–240)	980.5	0.002
Total motility (%)	60 (40–80)	30 (5–60)	890	0.003
Progressive motility (%)	35 (15–55)	15 (0–40)	865	0.001

Participants with elevated body mass index (≥23 kg/m²) demonstrated lower median sperm concentration, total sperm count, total motility, and progressive motility compared with those with normal body mass index, whereas semen volume did not differ significantly between groups (adjusted p = 0.331).

Similarly, participants with increased waist circumference (≥90 cm) exhibited lower median sperm concentration, total sperm count, and motility-related parameters compared with those with normal waist circumference. After adjustment for multiple comparisons, progressive motility remained the most consistently associated parameter (adjusted p = 0.001), while differences in semen volume were not statistically significant (adjusted p = 0.165).

**Figure 1:**
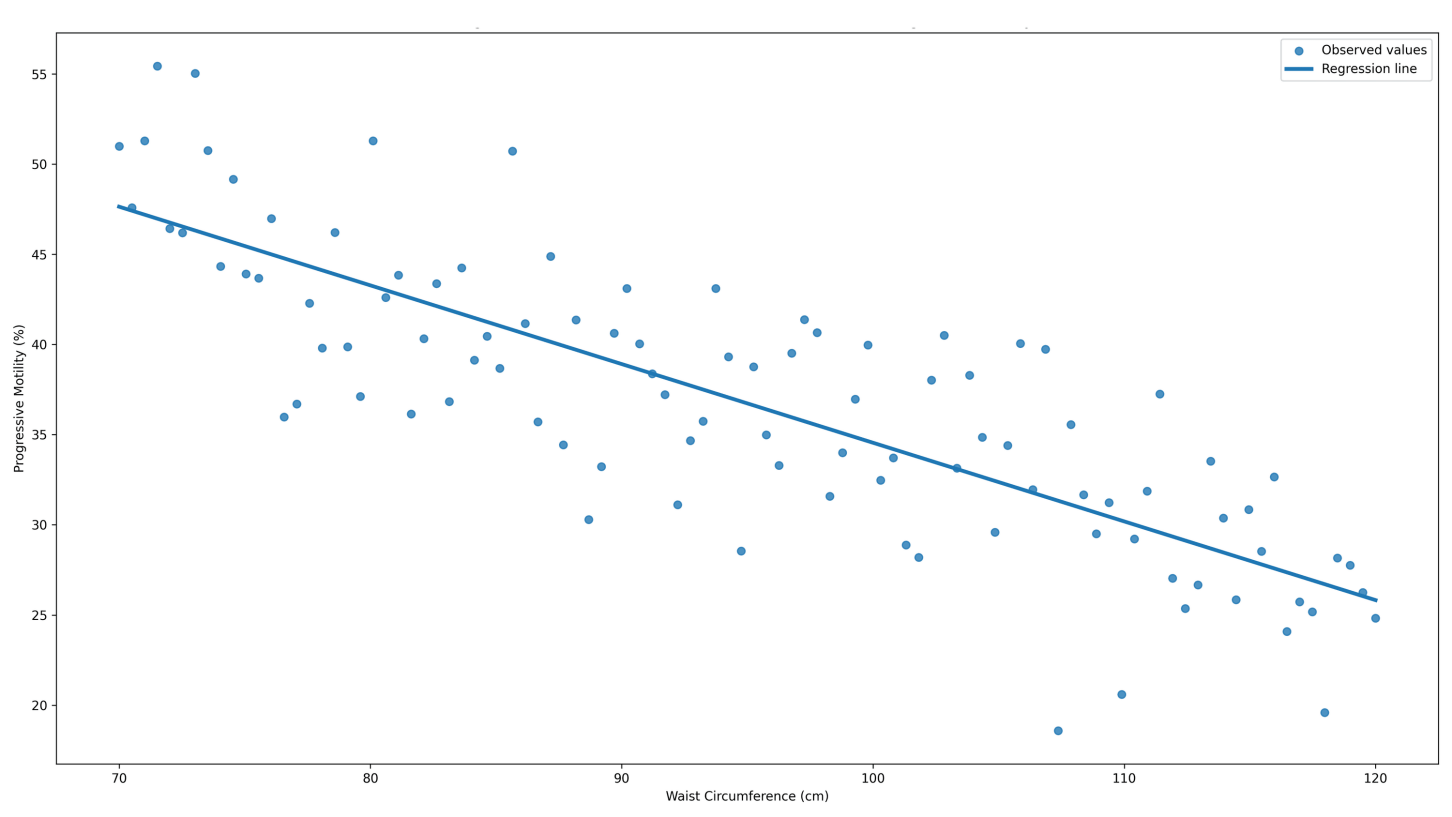
Correlation between waist circumference and progressive sperm motility Correlation between waist circumference and progressive sperm motility. Scatter plot illustrating the inverse association between waist circumference (cm) and progressive sperm motility (%). Spearman’s rank correlation demonstrated a moderate negative correlation (r = −0.421), which remained statistically significant after Holm–Bonferroni adjustment (adjusted p = 0.00004).

Spearman’s correlation analysis demonstrated a significant inverse association between waist circumference and progressive sperm motility (r = −0.421, adjusted p < 0.001), as illustrated in Figure 1. 

Association of semen parameters with WHR and WHtR

As presented in Tables 5, 6, participants with elevated waist-hip ratio and waist-height ratio demonstrated lower median values of total sperm count and motility-related parameters compared with those with normal indices. Across both measures of central adiposity, progressive motility showed the most consistent and pronounced reduction.

**Table 5:** Association of semen parameters with waist–hip ratio using non-parametric analysis Values are expressed as median (interquartile range). Group comparisons were performed using the Mann–Whitney U test. Spearman’s rank correlation coefficient (r) is used for correlation analysis. †P values were adjusted for multiple comparisons using the Holm–Bonferroni method. Statistical significance is defined as adjusted p < 0.05.

Parameter	Median (IQR) - Waist–hip ratio <0.90	Median (IQR) - Waist–hip ratio ≥0.90)	U value	Adjusted p value†
Semen volume (mL)	3.5 (2.5–5.0)	2.5 (1.5–4.0)	1176.5	0.015
Sperm concentration (million/mL)	80 (40–120)	50 (10–100)	1149.0	0.015
Total sperm count (million/ejaculate)	300 (150–450)	120 (20–250)	1275.0	0.002
Total motility (%)	65 (45–80)	35 (5–60)	1238.0	0.004
Progressive motility (%)	45 (25–65)	15 (0–40)	1302.0	0.001

**Table 6:** Association of semen parameters with waist–height ratio using non-parametric analysis Values are expressed as median (interquartile range). Group comparisons were performed using the Mann–Whitney U test. Spearman’s rank correlation coefficient (r) is used for correlation analysis. †P values were adjusted for multiple comparisons using the Holm–Bonferroni method. Statistical significance is defined as adjusted p < 0.05.

Parameter	Median (IQR) - Waist–height ratio <0.50	Median (IQR) -Waist–height ratio ≥0.50	U value	Adjusted p value†
Semen volume (mL)	3.0 (2.0–4.5)	2.5 (1.5–4.0)	1338.0	0.290
Sperm concentration (million/mL)	70 (30–120)	50 (10–100)	1479.5	0.095
Total sperm count (million/ejaculate)	260 (130–400)	150 (30–250)	1559.0	0.045
Total motility (%)	60 (40–80)	40 (10–60)	1492.0	0.095
Progressive motility (%)	35 (15–55)	20 (0–40)	1547.5	0.045

Differences in semen volume and sperm concentration were less uniform and did not reach statistical significance in several comparisons after adjustment for multiple testing, suggesting that motility-related parameters may be more sensitive to central obesity indices than volume-based measures.

Spearman's rank correlations between anthropometric indices and semen parameters are presented in Table 7. 

**Table 7:** Spearman rank correlation between anthropometric indices and semen parameters BMI = body mass index; WC = waist circumference; WHR = waist–hip ratio; WHtR = waist–height ratio. Correlation coefficients represent Spearman’s rank correlation (r), two-tailed. †P values were adjusted for multiple correlations using the Holm–Bonferroni method. Correlation strength was interpreted as weak (r < 0.30) or moderate (r = 0.30–0.50). Statistical significance was defined as adjusted p < 0.05.

Semen parameter	BMI (r)	WC (r)	WHR (r)	WHtR (r)	Adjusted p value†
Semen volume	−0.20	−0.25	−0.34	−0.23	0.018
Sperm concentration (million/mL)	−0.34	−0.35	−0.26	−0.33	0.003
Total sperm count (million/ejaculate)	−0.39	−0.42	−0.35	−0.39	0.001
Total motility (%)	−0.39	−0.38	−0.32	−0.36	0.001
Progressive motility (%)	−0.43	−0.42	−0.37	−0.41	0.001

**Figure 2:**
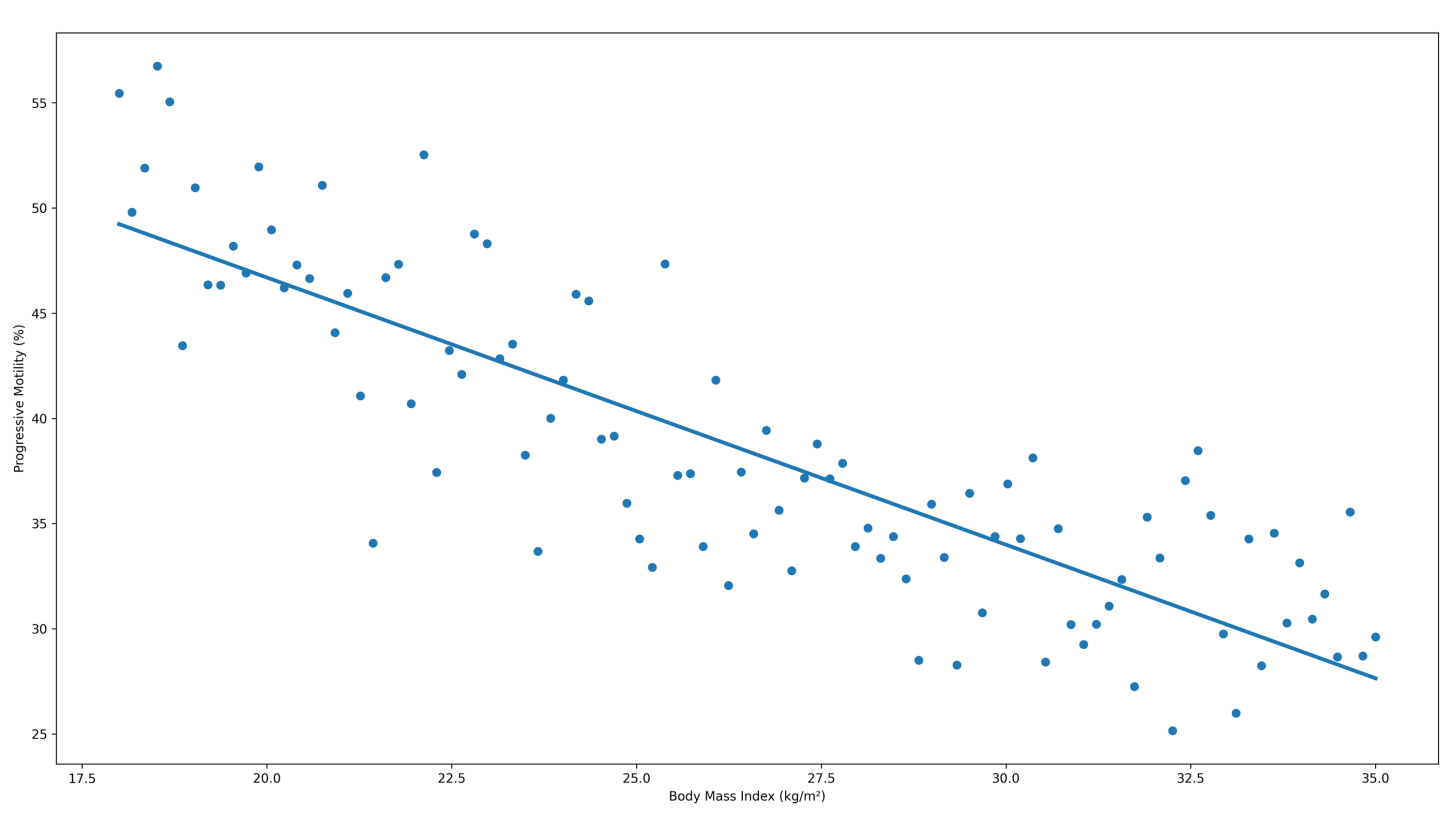
Relationship between body mass index and progressive sperm motility Scatter plot illustrating the association between body mass index and progressive sperm motility (%). The solid line represents a locally weighted trend line for visualization purposes. Correlation was assessed using Spearman’s rank correlation coefficient (r = −0.426). The association remained statistically significant after adjustment for multiple comparisons (adjusted p < 0.001).

**Figure 3:**
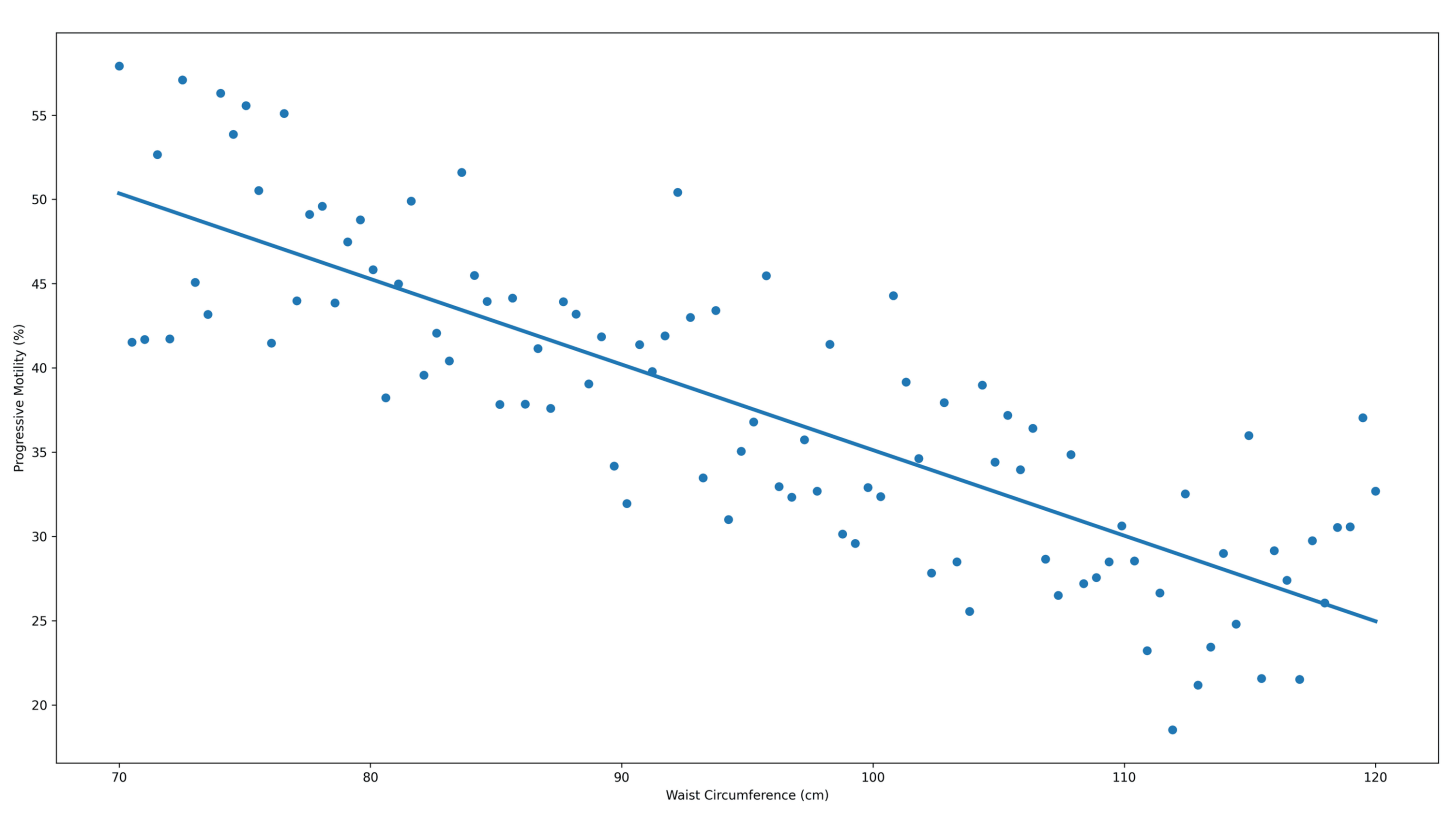
Relationship between waist circumference and progressive sperm motility Scatter plot illustrating the association between waist circumference (cm) and progressive sperm motility (%). The solid line represents a locally weighted trend line for visualization purposes. Correlation was assessed using Spearman’s rank correlation coefficient (r = −0.424). The association remained statistically significant after adjustment for multiple comparisons (adjusted p < 0.001).

**Figure 4:**
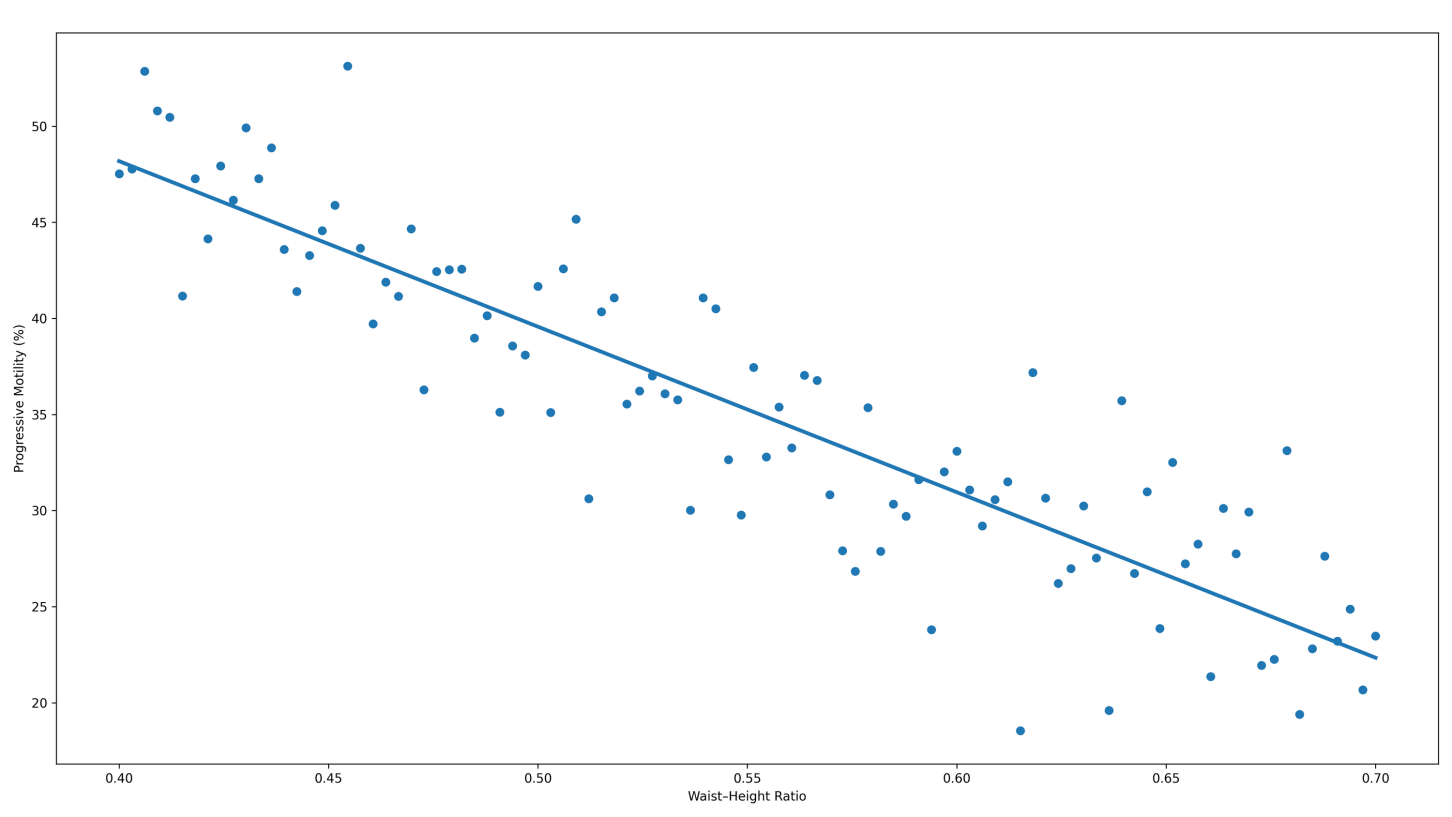
Relationship between waist–height ratio and progressive sperm motility Scatter plot illustrating the association between waist–height ratio and progressive sperm motility (%). The solid line represents a locally weighted trend line for visualization purposes. Correlation was assessed using Spearman’s rank correlation coefficient (r = −0.410). The association remained statistically significant after adjustment for multiple comparisons (adjusted p < 0.001).

To visually illustrate the associations between anthropometric indices and progressive sperm motility, scatter plots depicting individual observations and non-parametric trend lines are presented in Figures 2-4.

## Discussion

The findings of this study demonstrate that both general and central adiposity are inversely associated with key semen parameters, particularly sperm concentration, total sperm count, and motility-related outcomes. Among all semen characteristics examined, progressive motility consistently exhibited the moderate inverse association across body mass index (BMI), waist circumference (WC), waist-hip ratio (WHR), and waist-height ratio (WHtR). In contrast, semen volume showed weaker and less consistent associations with most anthropometric indices, with the notable exception of waist-hip ratio, for which a significant inverse association was observed (Table 3-7). This suggests that central fat distribution, as captured by WHR, may influence accessory gland function or ejaculate composition differently than overall or abdominal adiposity. 

These findings suggest that obesity may preferentially affect spermatogenic efficiency and sperm functional competence, rather than ejaculate volume. The preservation of semen volume despite impairment of sperm concentration and motility supports the concept that accessory gland secretion may be less susceptible to adiposity-related alterations compared with testicular and post-testicular processes involved in sperm production and maturation.

Comparison with previous literature: BMI and semen quality

The relationship between BMI and semen quality has been extensively studied, with mixed results reported across populations. Data from the LIFE study demonstrated that higher BMI and WC were associated with lower sperm concentration and total sperm count, findings that closely align with the present results [9]. Similarly, several observational studies and systematic reviews have reported inverse associations between BMI and semen parameters, particularly sperm count and motility [7,11].

Conversely, some investigations have failed to detect significant associations between BMI and semen quality, highlighting the heterogeneity of findings in this field [17]. Differences in study design, population characteristics, statistical methodology, and definitions of obesity may account for these discrepancies. Importantly, the present study used Asian-specific BMI cut-offs, which may be more appropriate for identifying metabolic risk in this population [12], thereby improving sensitivity for detecting reproductive associations.

Central obesity indices are stronger predictors

A key contribution of the present study is the demonstration that central obesity indices showed a moderate association with semen quality compared to BMI alone. Waist circumference, WHR, and WHtR were all inversely associated with sperm concentration, total sperm count, and motility parameters, with progressive motility showing the most pronounced reduction.

These findings are consistent with earlier reports suggesting that fat distribution may be more relevant to male reproductive health than overall body mass. Fejes et al. first highlighted the potential role of body fat distribution in semen quality, reporting moderate associations for WHR than BMI [18]. Subsequent studies confirmed that central obesity indices were more closely related to impaired semen parameters and hormonal disturbances [18,19,10]. The present study extends this evidence by demonstrating consistent associations across multiple central obesity measures within the same cohort.

Biological plausibility and mechanistic pathways

The observed associations between adiposity and semen quality are biologically plausible and supported by established pathophysiological mechanisms. Obesity is associated with endocrine dysregulation, including reduced testosterone levels and altered testosterone-to-estradiol ratios due to increased aromatization in adipose tissue [8]. These hormonal changes can adversely affect spermatogenesis and sperm maturation.

Additionally, obesity is linked to insulin resistance, chronic low-grade inflammation, and oxidative stress, all of which may impair testicular function and sperm quality [20]. Sperm motility, particularly progressive motility, is highly dependent on mitochondrial energy production and membrane integrity, making it especially vulnerable to oxidative damage. This may explain why progressive motility consistently emerged as the most sensitive semen parameter in relation to adiposity in the present study.

Occupational and environmental exposures represent additional factors that may influence male reproductive health. Exposure to heat stress, industrial chemicals, pesticides, heavy metals, air pollution, and endocrine-disrupting compounds has been associated with impaired spermatogenesis and reduced sperm motility. Although individuals with known major clinical risk factors were excluded, detailed occupational exposure assessment was not formally quantified in the present study and may represent a source of residual confounding. Future research incorporating standardized environmental and occupational exposure metrics would provide a more comprehensive evaluation of male infertility determinants.

Clinical relevance of WHR and WHtR

The moderate associations observed for WHR and WHtR merit particular attention. WHR has long been recognized as a marker of body fat distribution and metabolic risk [21], while WHtR has been proposed as a superior screening tool for cardiometabolic risk compared with BMI and WC [14]. The present findings suggest that WHtR may also be a useful indicator of reproductive risk, supporting its inclusion in clinical assessment of infertile men.

Given the simplicity and low cost of measuring WC, WHR, and WHtR, incorporation of these indices into routine infertility evaluation may enhance risk stratification and counseling. The findings also reinforce the importance of targeting central obesity, rather than focusing solely on overall weight reduction.

Consistency with interventional evidence

Although the present study is cross-sectional, its findings are supported by interventional evidence suggesting potential reversibility of obesity-related reproductive impairment. Weight loss has been associated with improvements in semen parameters and reproductive hormone profiles in severely obese men [15]. These observations lend further plausibility to the associations identified in the present analysis and underscore the potential clinical relevance of lifestyle modification in this context.

Methodological strengths

Several strengths enhance the credibility of this study. First, semen analysis was conducted using standardized procedures in accordance with WHO laboratory guidelines [16]. Second, anthropometric indices were defined using population-appropriate cut-offs, including Asian-specific BMI and WC thresholds [12,13]. Third, the use of non-parametric statistical methods appropriately accounted for skewed data distributions, while adjustment for multiple comparisons reduced the risk of false-positive findings [22,23]. Finally, the inclusion of multiple anthropometric measures allowed a nuanced evaluation of both general and central obesity.

Limitations

Certain limitations should be acknowledged. The cross-sectional design precludes causal inference and does not allow assessment of temporal relationships between obesity and semen quality. The study population consisted of male partners of infertile couples, which may limit generalizability to the broader male population. Hormonal and metabolic parameters were not assessed, restricting mechanistic interpretation. Additionally, lifestyle factors such as diet and physical activity were not formally quantified and may have contributed to residual confounding.

Implications for future research

Future studies should adopt longitudinal and interventional designs to clarify causality and assess the impact of weight reduction on semen quality and fertility outcomes. Integration of hormonal, metabolic, and oxidative stress markers would provide deeper mechanistic insight. Larger, population-based studies are also needed to confirm the generalizability of these findings and to explore potential ethnic and regional differences. Finally, evaluation of assisted reproductive outcomes in relation to male anthropometry may further elucidate the clinical significance of obesity in reproductive medicine.

## Conclusions

This study identifies significant associations between both general and central anthropometric indices of obesity and semen quality among male partners of infertile couples without identifiable clinical risk factors for infertility other than adiposity. Measures of central obesity demonstrated more consistent relationships with semen parameters than body mass index alone, with progressive motility emerging as the most sensitive parameter across anthropometric indices. The principal contribution of this study lies in its comprehensive and simultaneous evaluation of multiple indices of general and central adiposity within a well-defined population, using standardized semen analysis protocols and appropriate non-parametric statistical methods. Given the cross-sectional design, causal inferences cannot be drawn, and the observed associations should be interpreted cautiously.

From a clinical and research perspective, these findings highlight the potential relevance of central obesity assessment in the evaluation of male infertility and support further investigation into adiposity as a modifiable correlate of semen quality. Prospective and interventional studies are warranted to clarify underlying mechanisms and to determine whether targeted reduction of central adiposity is associated with improvements in semen quality and reproductive outcomes.
